# Decoding PANoptosis in Gout: Signature Gene Identification and Immune Infiltration Profiling

**DOI:** 10.1111/1756-185X.70344

**Published:** 2025-07-04

**Authors:** Junjie Cao, Aifang Li, Gaiying Luo, Zhen Wu, Yuan Liu

**Affiliations:** ^1^ Department of Laboratory Medicine Xi'an Fifth Hospital Xi'an Shaanxi Province China; ^2^ Department of Laboratory Medicine Xi'an Chest Hospital Xi'an Shaanxi Province China; ^3^ Department of Rheumatology (Unit 6) Xi'an Fifth Hospital Xi'an Shaanxi Province China

**Keywords:** bioinformatics, gout, immune infiltration, PANoptosis

## Abstract

**Background:**

Gout is an inflammatory disorder triggered by the deposition of monosodium urate (MSU) crystals in joints and periarticular tissues. PANoptosis, a recently identified form of inflammatory cell death, remains uncharacterized in gout pathogenesis. This study aims to identify PANoptosis‐related genes that may drive gout progression.

**Methods:**

Gout‐related datasets, including the human cohort (GSE160170) and murine model (GSE190138), were retrieved from the Gene Expression Omnibus (GEO) database. Differentially expressed genes (DEGs) were screened using thresholds of |log_2_ fold change (FC)| ≥ 1 and adjusted *P*‐value < 0.05. PANoptosis‐related biomarkers were identified through the combined use of MCODE and cytoHubba algorithms in Cytoscape. Least Absolute Shrinkage and Selection Operator (LASSO) regression was applied to select hub genes. Subsequently, we performed single—sample gene set enrichment analysis (ssGSEA) for the hub genes, analyzed the infiltration levels of immune cells, constructed a miRNA–mRNA–transcription factor (TF) regulatory network, and identified potential therapeutic drugs via the DSigDB database and the Coremine Medical database. Finally, the expression of the diagnostic gene was validated by real‐time quantitative reverse transcription polymerase chain reaction (RT‐qPCR).

**Results:**

The PANoptosis‐associated gene *SOCS3* was identified via integrative bioinformatics screening. Enrichment analysis and immune infiltration assessment revealed its involvement in gout pathogenesis through pathways linked to inflammation and cell death, with significant correlations observed with specific immune cell subsets. Clinical validation via RT‐qPCR confirmed a strong consistency between SOCS3 expression levels in gout patients and computational predictions.

**Conclusion:**

We identified the hub gene *SOCS3* in gout and elucidated its mechanistic roles by integrated bioinformatics analysis, machine learning approach, and clinical validation, providing critical insights for advancing diagnostic biomarkers and therapeutic strategies in gout management.

## 1 Introduction

Gout is a metabolic‐inflammatory disorder caused by the deposition of monosodium urate (MSU) crystals in joints or periarticular tissues, clinically manifesting as gouty arthritis (GA), urate nephrolithiasis, or chronic gouty kidney disease [1]. The global prevalence of gout exhibits significant geographical variation, ranging from 3% to 5% in developed countries, while China reports a lower prevalence of 1.6%, though both regions show a persistent upward trend [2]. Currently, the gold standard for diagnosing gout is the presence of MSU crystals in synovial fluid or tophi using polarized light microscopy, but the detection of MSU crystals is subject to limitations in medical conditions, examination conditions, and the technician's skill, resulting in suboptimal sensitivity for early‐stage diagnosis [3].

Cell death plays a pivotal role in both physiological and pathological processes. Emerging evidence indicates that distinct cell death modalities are associated with the clinical progression of gout, where immune cells release cytokines and chemokines through necroptosis, pyroptosis, or other forms of cell death. This process amplifies immune cell recruitment to joints and exacerbates inflammatory cascades [4]. Key inflammatory mediators such as tumor necrosis factor (TNF)‐α and interleukin (IL)‐1β, which are critical regulators of apoptosis, necroptosis, and pyroptosis, are predominantly secreted by monocytes. These molecules promote inflammatory cell infiltration in gout patients [5, 6].

PANoptosis, a novel form of programmed cell death, integrates features of pyroptosis, apoptosis, and necroptosis, regulating disease progression through the formation of a multimolecular complex called the PANoptosome. Its core mechanism involves synergistic interactions of key molecules (e.g., ZBP1, NLRP3, Caspase family proteins) that ultimately drive inflammatory factor release and cell death [7]. The pathological hallmark of GA lies in the inflammatory response triggered by MSU crystal deposition. MSU crystals activate the NLRP3 inflammasome, triggering Caspase‐1‐dependent pyroptosis and subsequent release of pro‐inflammatory cytokines like IL‐1β [8, 9]. PANoptosis may exacerbate inflammatory signaling cascades and promote joint damage by integrating pyroptosis with other cell death modalities (e.g., apoptosis‐related Caspase‐8 or necroptosis‐associated MLKL) [10]. Therefore, investigating PANoptosis mechanisms in gout could facilitate the discovery of biomarkers for early diagnosis and targeted therapeutic interventions.

This study aims to investigate the relationship between PANoptosis and gout. By analyzing the gout data retrieved from the Gene Expression Omnibus (GEO) database, bioinformatics analysis was used to screen differentially expressed genes (DEGs) related to PANoptosis in gout. A protein–protein interaction (PPI) network was constructed to identify topologically central hub genes, ultimately screening for critical genes. The least absolute shrinkage and selection operator (LASSO) regression model was utilized in machine learning to identify hub genes. Gene set enrichment analysis (GSEA) was performed to identify potential signaling pathways. Correlation analysis was performed between PANoptosis‐related biomarkers and immune cell infiltration. Subsequently, relevant miRNAs and transcription factors (TFs) were predicted through online databases, and a miRNA–mRNA–TF network was constructed using Cytoscape software. The DSigDB database and the Coremine Medical database were adopted to screen for drugs targeting biomarkers. This provides potential biomarkers and regulatory pathways for the diagnosis and treatment of gout.

## 2 Materials and Methods

### 2.1 Study Design

The “GEOquery” package in R was employed to download gene expression datasets GSE160170 and GSE190138 from the GEO database. The GSE160170 dataset comprised peripheral blood mononuclear cell (PBMC) samples from 6 healthy controls and 6 gout patients, while GSE190138 included samples from 9 control mice and 9 gout model mice. Probe IDs were converted to gene symbols using the “idmap3” package based on platform annotation files. Differential expression analysis was performed with the “limma” package, applying thresholds of adjusted *P*‐value < 0.05 and |log_2_ fold change (FC)| ≥ 1 to identify significant DEGs. Volcano plots visualizing DEGs were generated using the “ggplot2” package. A total of 711 PANoptosis‐related genes were curated from literature sources, including 681 apoptosis‐associated genes, 6 necroptosis‐related genes, and 24 pyroptosis‐linked genes [11]. Gene Ontology (GO) and Kyoto Encyclopedia of Genes and Genomes (KEGG) enrichment analysis and PPI network analysis were performed on the common DEGs using online databases. Hub genes were prioritized using the MCODE and cytoHubba algorithms. The relative abundance of 28 distinct immune cell subsets was quantified using single‐sample Gene Set Enrichment Analysis (ssGSEA) to characterize immune infiltration patterns within the tissue microenvironment. miRNAs and TFs related to the hub genes were predicted through online databases, and a miRNA–mRNA–TF regulatory network was constructed using Cytoscape software. Hub gene‐associated drugs were predicted using the DSigDB database and the Coremine Medical database. PBMCs from gout patients and healthy individuals were collected, and the expression level of hub genes was verified by RT‐qPCR. The flowchart is shown in Figure S1.

### 2.2 Screening of Differentially Expressed Genes

Firstly, the “affy” package in the R software program was used for background correction, normalization, and log_2_ transformation of the gout datasets. The Limma package was used to identify DEGs in gout, with the criteria set as: |log_2_ Fold change (FC)| ≥ 1, and *P*‐value < 0.05. A total of 711 PANoptosis‐related genes were curated from literature sources, including 681 apoptosis‐associated genes, 6 necroptosis‐related genes, and 24 pyroptosis‐linked genes [11].

### 2.3 Functional Enrichment Analysis

The Database for Annotation, Visualization and Integrated Discovery (DAVID, https://david.ncifcrf.gov/) was used for GO analysis. To elucidate the functional implications of DEGs, GO enrichment analysis was performed using the DAVID tool (version 6.8), which interrogates the GO database to annotate biological processes, cellular components, and molecular functions. After downloading the GO analysis data, the “ggplot2” package was used to generate bubble charts. The KEGG Orthology Based Annotation System (KOBAS version 3.0, http://kobas.cbi.pku.edu.cn) was used for pathway analysis. The DAVID tool was used to explore the pathways enriched by DEGs and the biological significance behind them. After downloading the pathway analysis data, the “ggplot2” package was used to generate bubble charts. Subsequently, GSEA was performed to reveal the specific functions of each gene. The significance threshold was set at an adjusted *P*‐value < 0.05. In this study, based on the intersection of DEGs from gout and genes related to PANoptosis, a common set of genes was subjected to GO and KEGG analysis.

### 2.4 Construction of Protein–Protein Interaction (PPI) Network

The PPI network was constructed using the String database (version 11.5; www.string‐db.org) with an interaction score set to 0.400. The Cytoscape software was utilized to build this PPI network.

### 2.5 Hub Gene Screening

The PPI network was imported into the Cytoscape software to identify key functional modules and hub genes that play significant roles. The Molecular Complex Detection (MCODE) plugin was employed to recognize key functional modules; the identification criteria were: node degree ≥ 2, node score ≥ 0.2, K‐core ≥ 2, and maximum depth = 100. The top 10 hub genes were identified in the PPI network using the Maximal Clique Centrality (MCC) algorithm of the Cytohubba plugin. Ultimately, the intersection of genes from the MCODE key modules and the cytoHubba key genes was determined as the hub genes.

### 2.6 LASSO Regression Model

The R package “glmnet” was utilized to further screen candidate genes for gout diagnosis through LASSO‐Cox regression analysis. For the GSE160170 dataset, the optimal *λ* (lambda) value was 0.07, while for the GSE190138 dataset, *λ* was set to 0.06. The intersection of genes selected from both datasets was identified as key genes.

### 2.7 Immune Infiltration Analysis

The infiltration of 28 immune cell types was analyzed using ssGSEA with the R package “GSVA”. The correlations among immune cells were visualized using heatmaps generated by the R package “corrplot” *p < 0.05, **p < 0.01, ***p < 0.001.

### 2.8 miRNA–mRNA–TF Regulatory Network in Gout

Three online miRNA databases, miRWalk, miRNANet, and miRTarBase, were used to predict miRNAs. The TRRUST database was used to predict associated TFs for the target mRNAs. Using the “merge” function in Cytoscape, a regulatory network of miRNA–mRNA–TF interactions was constructed.

### 2.9 Real‐Time Quantitative Reverse Transcription Polymerase Chain Reaction (RT‐qPCR)

A total of 10 gout patients and 10 healthy controls were recruited from the Xi'an Fifth Hospital. The gout patients met the 2015 EULAR/ACR classification criteria for gout. The PBMCs from gout patients and healthy individuals were collected. Total RNA was isolated from cells using the TRIzol reagent (Invitrogen) according to the manufacturer's instructions. RT‐qPCR was performed using SYBR Green PCR Master Mix (Bio‐Rad Laboratories) on a MyiQ Single‐Color Real‐time PCR Detection System (Bio‐Rad Laboratories). Sequence‐specific primers for the indicated genes were synthesized by Sangon Biotech and are listed in Table S1. The study was approved by the Research Ethics Committee of the Xi'an Fifth Hospital (2023–74).

### 2.10 Predicting Drugs for Key Genes

To identify potential therapeutics targeting the key genes, we performed drug enrichment analysis using the DSigDB database. The gene signature was uploaded to the Enrichr platform (https://maayanlab.cloud/Enrichr/) to evaluate associations with drug‐induced gene expression profiles. Significantly small‐molecule drug candidates were filtered by an adjusted *p*‐value < 0.05. The top 10 drugs ranked by the Combined Score were further analyzed based on their known mechanisms of action and clinical relevance to the disease context. Additionally, the Coremine Medical database (https://www.pubgene.com/coremine‐medical/) was utilized to query traditional Chinese medicines (TCMs) statistically associated with the key genes (*p*‐value < 0.05).

### 2.11 Statistical Analysis

All statistical analyses were conducted using R software (version 4.3.3) and GraphPad Prism 9 (GraphPad Software Inc., San Diego, CA, USA). Normality of continuous variables was assessed via the Shapiro–Wilk test. For intergroup comparisons: Normally distributed data were analyzed using independent two‐tailed Student's *t*‐tests, and non‐normally distributed data were evaluated with the Mann–Whitney U test. Spearman's rank correlation analysis was employed to assess variable associations. Statistical significance was defined as *p* < 0.05.

## 3 Results

### 3.1 Identification and Functional Enrichment Analysis of DEGs in Gout

**FIGURE 1:**
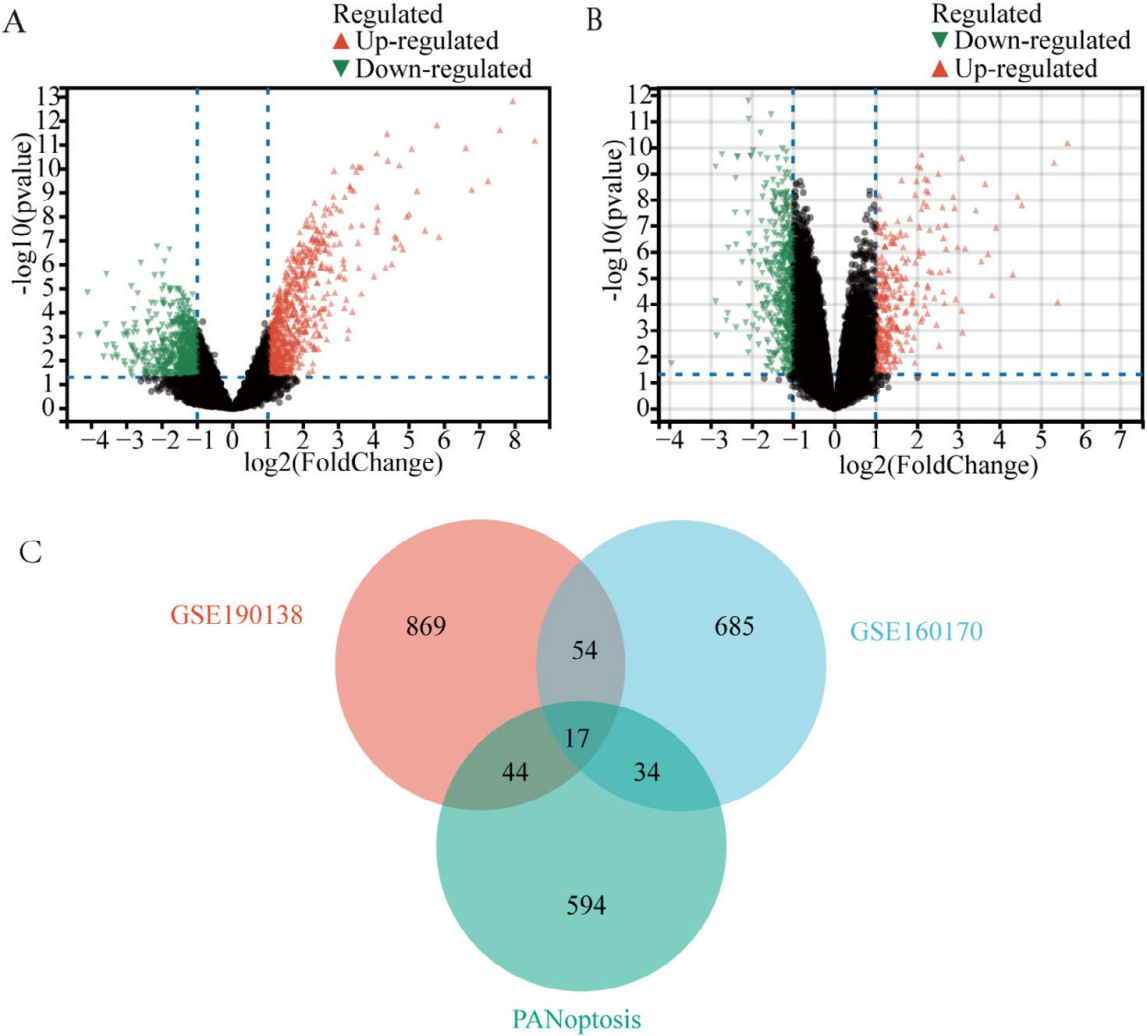
Identification of DEGs in Gout and common genes with PANoptosis. (A) The volcano map of gout dataset GSE190138. (B) The volcano map of Gout dataset GSE160170. Upregulated genes were marked in red; downregulated genes were marked in green. (C) The overlap genes of DEGs in GSE190138, GSE160170, and genes with PANoptosis via a Venn diagram.

In the GSE190138 dataset, 1469 DEGs were yielded, with 722 upregulated and 747 downregulated genes (Figure 1A). In the GSE160170 dataset, 790 DEGs were identified, comprising 303 upregulated and 487 downregulated genes (Figure 1B). Intersection analysis with 711 PANoptosis‐related genes revealed 17 overlapping genes (Figure 1C).

To further understand and study the biological functions of the DEGs related to PANoptosis, GO and KEGG enrichment analyses were performed on the above 17 genes. The results were shown in Figure S2. In the KEGG enrichment analysis, most genes were involved in the TNF signaling pathway, the NF‐kappa B signaling pathway, the IL‐17 signaling pathway, apoptosis, and necroptosis (Figure S2A). Biological Process (BP) was mainly enriched in pathways related to cell death (Figure S2B); Cellular Component (CC) was primarily enriched in the extracellular region, plasma membrane part, lysosomes, etc. (Figure S2C); Molecular Function (MF) were mainly enriched in the molecular function regulators, cytokine activity, cytokine receptor binding, etc. (Figure S2D).

### 3.2 Hub Gene Screening

**FIGURE 2:**
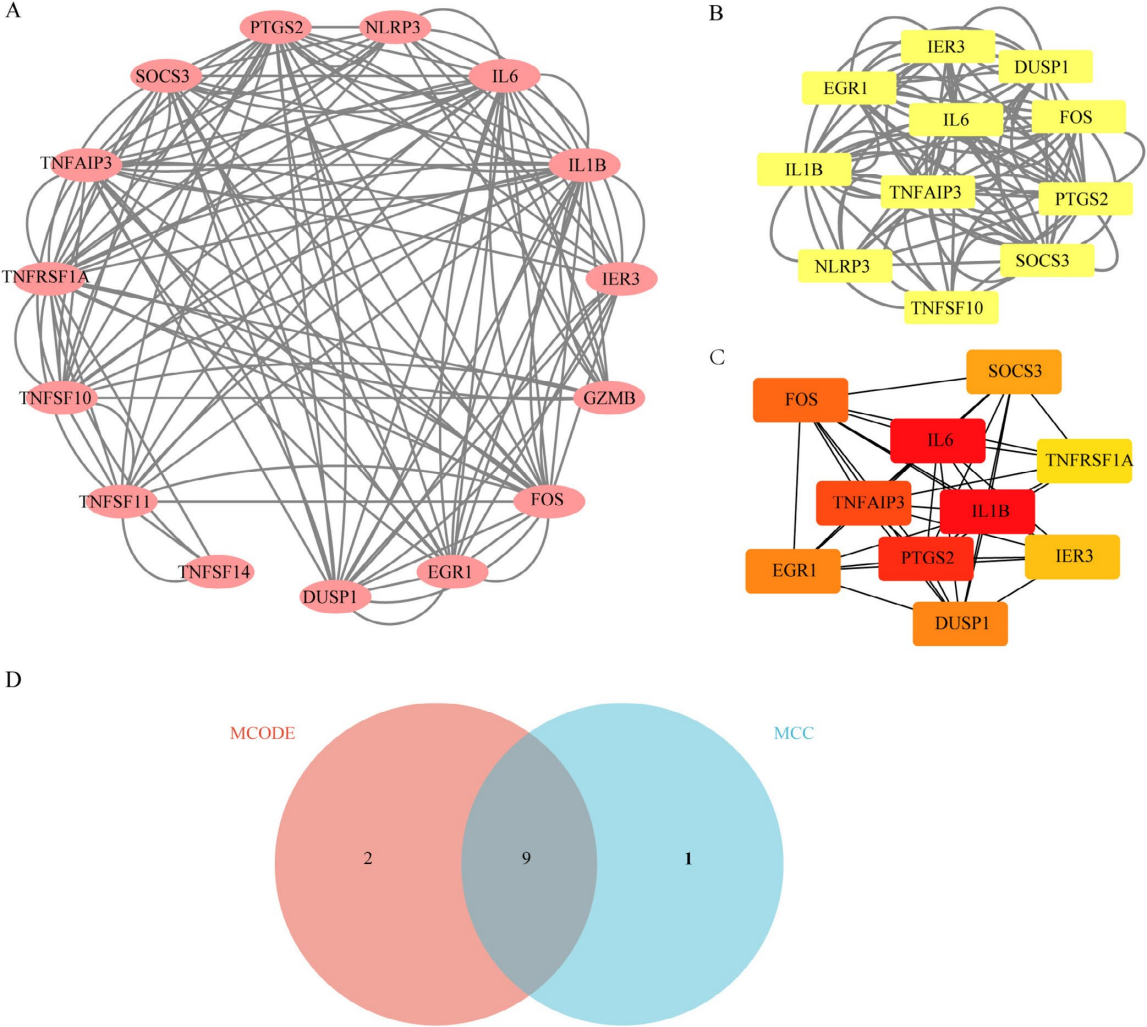
Protein–protein interaction (PPI) network and hub genes screening. (A) PPI network of common genes in Gout and PANoptosis. (B) 11 genes clustered in one module were exhibited by MCODE. (C) The top 10 hub genes identified using the MCC algorithm in the cytoHubba plugin. (D) Intersection of the MCODE key module genes and the MCC algorithm hub genes via a Venn diagram.

Based on the STRING database, interactions were found among 15 of the 17 DEGs. A visual PPI network diagram was established using the Cytoscape software (Figure 2A). To better understand the potential relationships between DEGs related to PANoptosis, the PPI network was further imported into the Cytoscape software to screen for key functional modules and hub genes that play important roles. One key module (containing 11 genes) was identified using the MCODE plugin (Figure 2B), which may represent important regulatory pathways for genes related to PANoptosis in gout. Second, the top 10 hub genes were identified using the MCC algorithm in the cytoHubba plugin (Figure 2C). Finally, the intersection of the MCODE key module genes and the cytoHubba key genes resulted in 9 hub genes (*PTGS2*, *SOCS3*, *TNFAIP3*, *IER3*, *DUSP1*, *FOS*, *EGR1*, *IL6*, and *IL1B*) (Figure 2D).

### 3.3 Machine Learning Screening for Gout Candidate Genes

**FIGURE 3:**
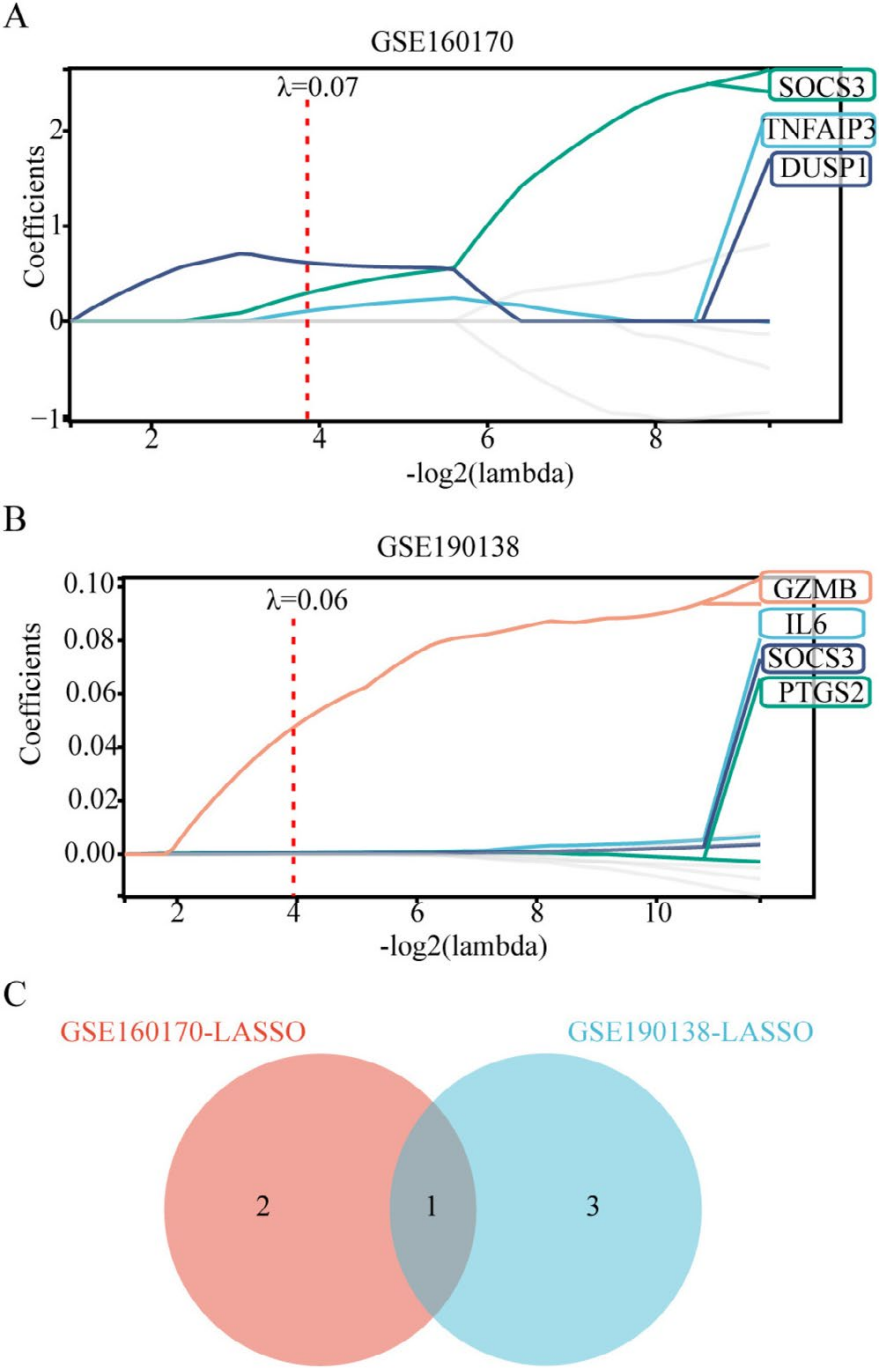
Machine learning screening for gout candidate genes. (A) Identification of biomarkers using the Lasso regression model in the GSE160170 dataset. (B) Identification of biomarkers using the Lasso regression model in the GSE190138 dataset. (C) Intersection of the LASSO candidate genes between GSE160170 and GSE190138 via Venn diagram.

The LASSO machine learning algorithm was employed to analyze the previously identified 9 genes in both the GSE160170 and GSE190138 datasets to screen candidate diagnostic genes for gout. In the GSE160170 dataset, the selected genes were *TNFAIP3, SOCS3*, and *DUSP1*, while the GSE190138 dataset yielded *IL6, PTGS2, SOCS3*, and *GZMB* (Figure 3A,B). The shared gene between both datasets was *SOCS3* (Figure 3C).

### 3.4 Expression of Key Genes and Gene Set Enrichment Analysis (GSEA)

**FIGURE 4:**
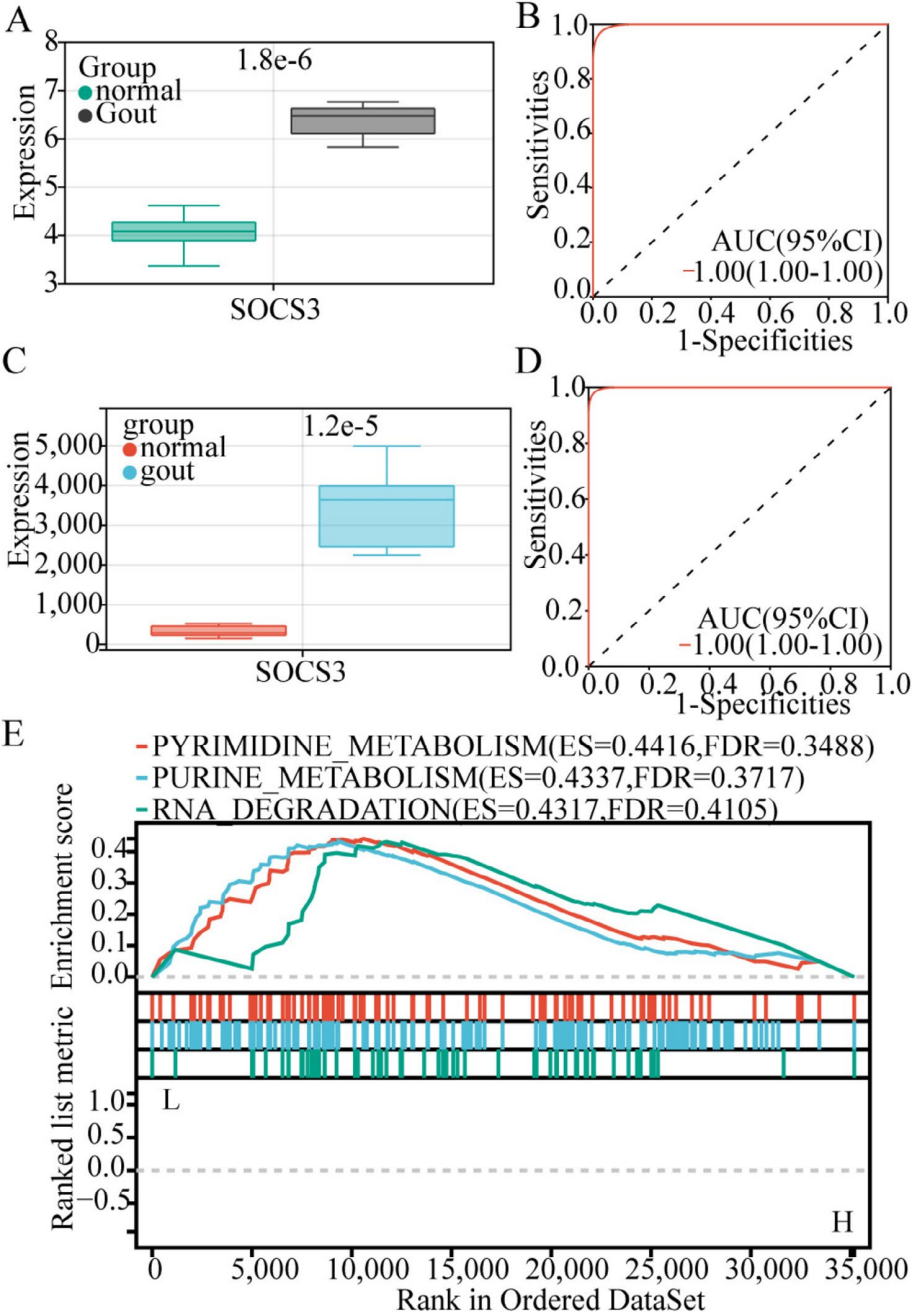
Expression of Key Genes and GSEA Analysis. (A, B) The expression of *SOCS3* and ROC curve validated in the GSE160170. (C, D) The expression of *SOCS3* and ROC curve was validated in the GSE190138. (E) GSEA analysis of *SOCS3*.

The expression of *SOCS3* was validated in the GSE160170 and GSE190138 datasets, respectively. Compared to the normal control group, *SOCS3* was significantly upregulated in the gout group (Figure 4A,C), and the area under the ROC curve (AUC) for both datasets reached 100% (Figure 4B,D). GSEA analysis revealed that *SOCS3* was mainly concentrated in the purine metabolism, pyrimidine metabolism, and RNA degradation pathways (Figure 4E).

### 3.5 Immune Infiltration Analysis

**FIGURE 5:**
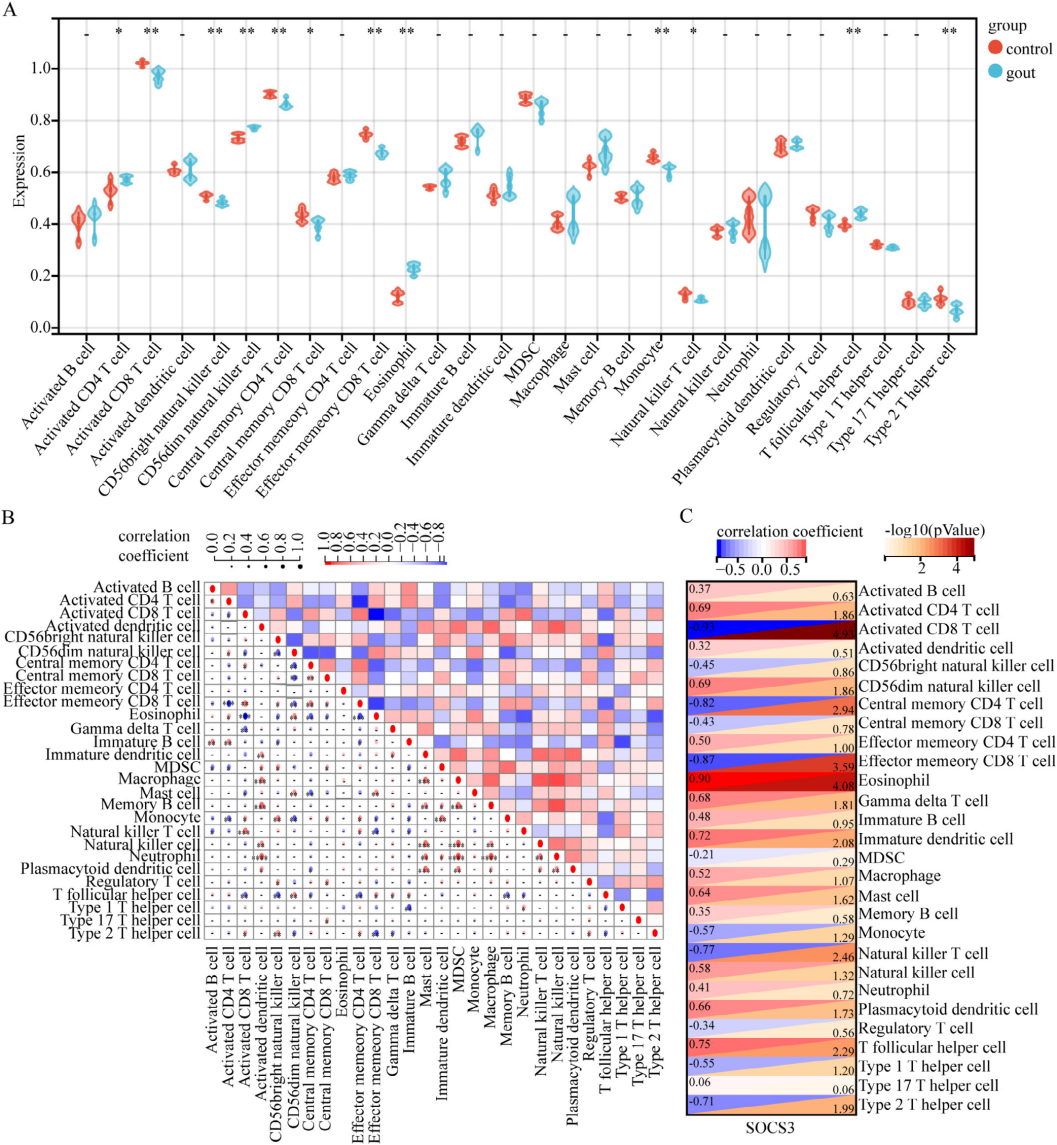
Immune cell infiltration analysis between gout and control. (A) Comparison of the proportions of 28 immune cell types between the gout group and the control group. (B) Association analysis of the 28 immune cell subtypes. Both horizontal and vertical axes demonstrate immune cell subtypes. (C) Correlation analysis between 28 immune cell types and *SOCS3*. *p < 0.05, **p < 0.01, ***p < 0.001.

Using ssGSEA, we characterized the infiltration patterns of 28 immune cell subpopulations in gout patients versus healthy controls. Significant differences in immune cell proportions were observed between the two groups. Specifically, 12 out of 28 immune cell subsets exhibited distinct variations, with activated CD4^+^ T cells, CD56^dim^ natural killer cells, eosinophils, and T follicular helper cells, showing marked upregulation in gout patients (Figure 5A). Next, we conducted a comprehensive correlation analysis among these 28 immune cell subtypes, revealing that many subtypes exhibited significant intercorrelations. (Figure 5B). Subsequently, we conducted an association analysis between the 28 immune cell subtypes and the *SOCS3*. The results indicated that *SOCS3* was negatively correlated with type 2 T helper cells, natural killer T cells, effector memory CD8^+^ T cells, central memory CD4^+^ T cells and activated CD8^+^ T cells, while it was positively correlated with T follicular helper cells, plasmacytoid dendritic cells, natural killer cells, mast cells, immature dendritic cells, gamma delta T cells, eosinophils, CD56^dim^ natural killer cells, and activated CD4^+^ T cells (Figure 5C). These findings suggest that these specific cell types may play a crucial role in the development of gout.

### 3.6 Construction of miRNA–mRNA–TF Network

**FIGURE 6:**
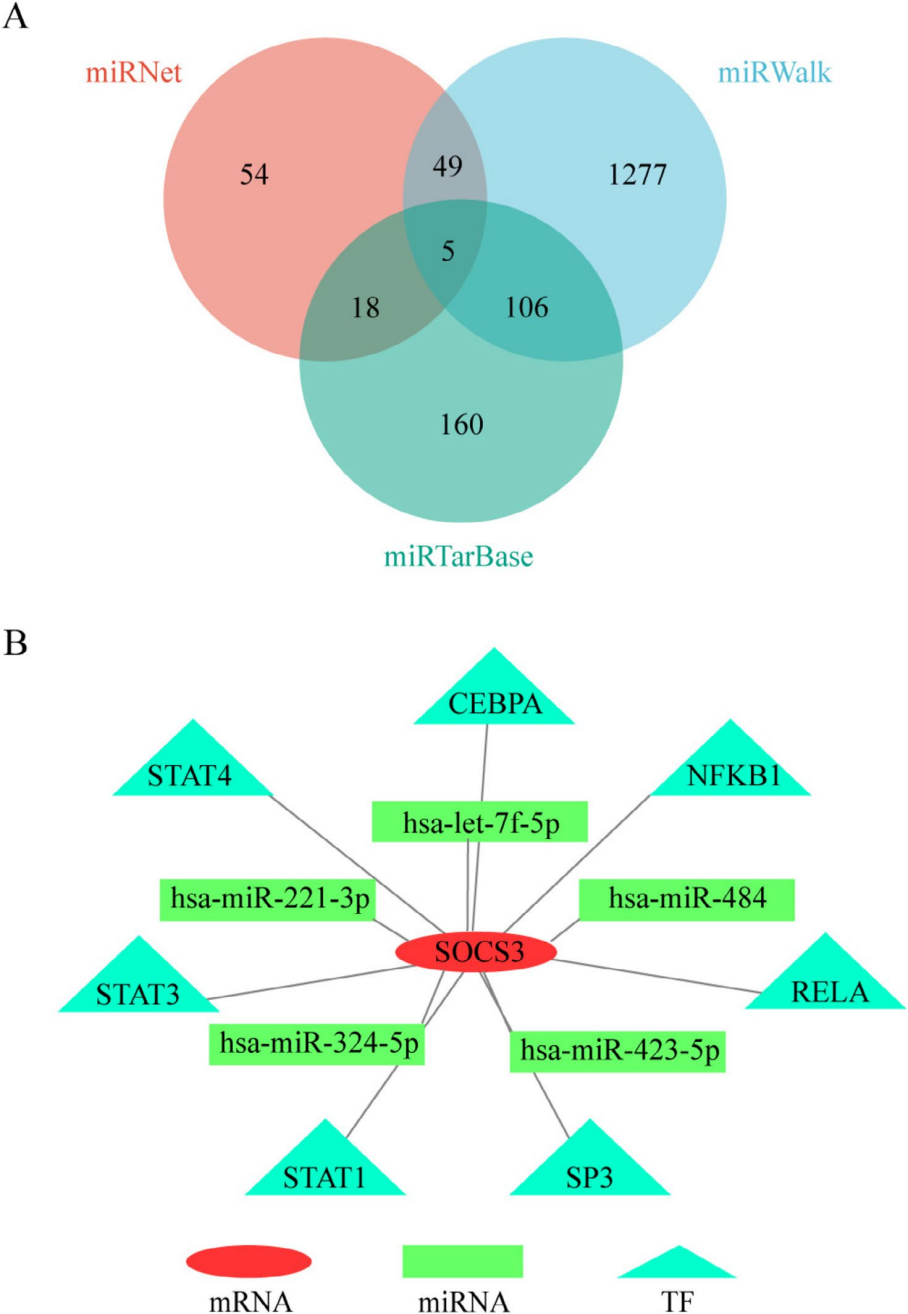
Construction of miRNA‐mRNA‐TF Network. (A) The overlapping miRNAs of miRWalk, miRNANet, and miRTarbase via Venn diagram. (B) The miRNA–mRNA–TF Network interactions by Cytoscape.

Three online miRNA databases (miRWalk, miRNANet, and miRTarBase) were applied to predict miRNAs, identifying five differentially expressed target miRNAs: *hsa‐let‐7f‐5p, hsa‐miR‐221‐3p, hsa‐miR‐324‐5p, hsa‐miR‐484*, and *hsa‐miR‐423‐5p* (Figure 6A).

In the TRRUST database, TFs related to the predicted *SOCS3* were identified. A total of 7 important TFs were finally screened out (*CEBPA*, *NFKB1*, *RELA*, *SP3*, *STAT1*, *STAT3*, *STAT4*). A miRNA–mRNA–TF network was constructed using the merge function in Cytoscape (Figure 6B).

### 3.7 Expression of Hub Genes in Clinical Samples

Compared with health control, the expression of *SOCS3* was upregulated in the gout group (Figure S3).

### 3.8 Screening for Potential Therapeutic Drugs

Using the DSigDB drug database for *SOCS3*‐targeted small‐molecule enrichment analysis, the top 10 candidates based on Combined Score (adjusted *p*‐value < 0.05) were identified (Table S2). Comprehensive analysis suggested that Isoeugenol and Rofecoxib may exert therapeutic effects on gout via *SOCS3* modulation. Additionally, 5 *SOCS3*‐related traditional Chinese herbs were screened from the Coremine Medical database (Table S3).

## 4 Discussion

Gout, a chronic metabolic‐inflammatory disorder, arises from dysregulated uric acid homeostasis driven by excessive purine catabolism, urate overproduction, and/or impaired renal urate excretion, culminating in persistent hyperuricemia (serum urate > 6.8 mg/dL) [12]. Monosodium urate (MSU) crystals form in synovial tissues when serum urate levels exceed saturation, triggering inflammation and pain [12]. The diagnostic gold standard remains the identification of MSU crystals in synovial fluid via joint aspiration; however, this invasive procedure demands specialized expertise and is not universally feasible in clinical practice [13]. Imaging tools like dual‐energy computed tomography (DECT) or ultrasound improve accuracy but face accessibility barriers [14]. Serum urate levels may normalize during acute flares due to inflammatory shifts, requiring post‐flare retesting for confirmation [15]. These limitations highlight the urgent need for non‐invasive biomarkers and diagnostic innovations to optimize gout management.

PANoptosis is a recently identified form of programmed inflammatory cell death characterized by its unique integration of molecular mechanisms from three classical cell death pathways: pyroptosis, apoptosis, and necroptosis. Its regulation relies on a multilayered macromolecular complex termed the PANoptosome, which triggers signaling cascades upon recognition of pathogen‐associated molecular patterns (PAMPs) or damage‐associated molecular patterns (DAMPs), ultimately leading to irreversible cell death. Unlike singular cell death pathways, the biological effects of PANoptosis cannot be fully explained by any individual mechanism alone. Instead, it operates through shared molecular components (e.g., caspase family proteins) and crosstalk between pathways [7, 16]. Current research suggests that PANoptosis may play a critical role in the pathogenesis and progression of gout by modulating inflammatory signaling pathways, immune cell functionality, and cell death mechanisms [8, 17, 18]. Therefore, elucidating the specific molecular mechanisms of PANoptosis in gout and identifying novel diagnostic biomarkers targeting the PANoptosis pathway could provide precision medicine strategies for gout patients.

Our study identified *SOCS3* as a central hub gene orchestrating gout‐associated inflammatory networks through integrated bioinformatics and machine learning approaches. *SOCS3* (Suppressor of Cytokine Signaling 3) is a critical member of the SOCS protein family and primarily functions to negatively regulate cytokine‐mediated signaling pathways, particularly the JAK–STAT pathway [19, 20]. Notably, recent mechanistic studies have demonstrated that *SOCS3* indirectly regulates PANoptosis‐associated genes (e.g., *CASP8*, *MLKL*) through suppression of STAT3 phosphorylation, thereby establishing a direct link to PANoptosis pathogenesis [21, 22]. Studies demonstrate that *SOCS3* inhibits PANoptosis through dual mechanisms: directly attenuating NF‐κB pathway activation (via reducing p65 phosphorylation and nuclear translocation) and indirectly modulating signaling mediators such as TLR4 and JAK2/STAT3, thereby downregulating inflammatory cytokines (IL‐6, TNF‐α) and cell death‐associated factors (Caspase‐8, RIPK3) [23]. These regulatory roles establish *SOCS3* as a multifunctional hub coordinating inflammatory responses and PANoptosis through crosstalk with core pathways, including JAK–STAT and NF‐κB. Recent studies have demonstrated that *SOCS3* exhibits significantly elevated expression in monocytes and inflammatory tissues during the acute phase of gout, showing marked differences compared to individuals with normal serum uric acid (SUA) levels or those with asymptomatic hyperuricemia (AH). Mechanistically, *SOCS3* may regulate the intensity of inflammatory responses by suppressing STAT3 phosphorylation, thereby limiting the signaling transduction of pro‐inflammatory cytokines such as IL‐6. This molecular regulatory mechanism modulates the amplification of inflammatory cascades and contributes to the pathological progression of gout [19, 24]. Collectively, these findings underscore SOCS3 as a pivotal biomarker influencing PANoptosis‐driven inflammatory severity during acute gout episodes.

Based on enrichment analysis findings, our study revealed a significant association between PANoptosis‐related genes and immune regulatory networks during the pathogenesis of gout. By systematically applying the ssGSEA method to conduct a panoramic characterization of the immune microenvironment in gout patients versus healthy controls, results demonstrated that 12 out of 28 evaluated immune cell subpopulations exhibited significant functional dysregulation. Further correlation analysis identified that the expression level of *SOCS3* showed significant negative correlations with five immune cell subsets, while displaying marked positive correlations with nine immune cell subsets. Notably, this differential association pattern suggests that *SOCS3* may act as a pivotal regulatory hub in gout‐related inflammatory pathology by modulating the functional states of specific immune cell populations. Studies have demonstrated that *SOCS3* enhances allergic responses by promoting the differentiation and activation of T helper 2 cells [25]. Its expression level is positively correlated with the pro‐inflammatory activity of mast cells and contributes to the pathogenesis of allergic diseases by regulating mast cell survival/degranulation and eosinophil activation/migration [25]. Current studies demonstrated that in inflammatory bowel disease (IBD), *SOCS3* acts on multiple cell types—including epithelial cells, macrophages, dendritic cells, neutrophils, and T cells—to repair mucosal damage and balance immune responses [26]. Studies have revealed that PANoptosis is primarily driven by molecular mechanisms in innate immune cells (e.g., macrophages, neutrophils, and dendritic cells). PANoptosis contributes to autoimmune diseases through chronic inflammation and aberrant immune activation. In conditions like systemic lupus erythematosus (SLE) and rheumatoid arthritis (RA), PANoptosis in innate immune cells (e.g., dendritic cells) may promote the release of self‐antigens, disrupt immune tolerance, and exacerbate inflammation via signaling pathways such as IL‐6/JAK‐STAT3 [16, 27]. These findings collectively highlight the pleiotropic immunomodulatory functions of *SOCS3* across diverse pathological contexts.

Our study constructed a miRNA–mRNA–TF regulatory network involving seven TFs (*CEBPA, NFKB1, RELA, SP3, STAT1, STAT3, STAT4*), five miRNAs (*hsa‐let‐7f‐5p*, *hsa‐miR‐221‐3p*, *hsa‐miR‐324‐5p*, *hsa‐miR‐484*, *hsa‐miR‐423‐5p*), and one mRNA (*SOCS3*). The NF‐κB signaling pathway plays a central role in gout‐associated inflammation. NFKB1 and RELA, key NF‐κB family members, directly regulate pro‐inflammatory cytokines IL‐1β and TNF‐α. Monocytes from gout patients exhibit heightened NF‐κB activity, closely linked to urate crystal‐triggered inflammatory cascades [28]. The STAT family (STAT1/STAT3/STAT4) mediates cytokine signaling in gout pathogenesis. IL‐6 activates STAT3 to promote neutrophil infiltration and joint inflammation, while *SOCS3*, a negative regulator of STAT3, is upregulated during gout flares, potentially mitigating inflammation by suppressing STAT3 signaling [22, 29]. STAT1 and STAT4 may also contribute to immune cell activation in gout pathology [30]. *Hsa‐let‐7f‐5p* is closely associated with inflammatory states in diseases. In macrophages, *hsa‐let‐7f‐5p* alleviates excessive inflammation by specifically downregulating the expression of interferon‐induced proteins Gbp2 and Gbp7 [31]. *Hsa‐miR‐221‐3p* exacerbates inflammatory responses by targeting and suppressing SOCS3, a negative regulator of the STAT3 signaling pathway, thereby relieving its inhibitory effect on STAT3 activation. This drives macrophage polarization toward the pro‐inflammatory M1 phenotype [32]. *Hsa‐miR‐423‐5p* is implicated in the inflammatory activation mechanisms of salt‐sensitive hypertension (SS). It participates in the KCNQ1OT1 long non‐coding RNA (lncRNA)‐mediated competitive endogenous RNA (ceRNA) network, where KCNQ1OT1 sequesters *hsa‐miR‐423‐5p* to attenuate its repression of downstream target genes. This process ultimately leads to vascular endothelial dysfunction and systemic inflammatory responses [33].

Based on the screening analysis of the DSigDB drug database, Isoeugenol and Rofecoxib were identified as potential small‐molecule drugs targeting *SOCS3* for gout treatment. Isoeugenol derivatives, such as ISO‐PC, significantly reduced serum uric acid levels by inhibiting xanthine oxidase (XOD) activity to suppress urate production and modulating renal urate transporters (e.g., ABCG2) to enhance uric acid excretion. Animal studies demonstrated that ISO‐PC exhibited protective effects against hyperuricemia‐induced hepatic and renal injuries [34]. Rofecoxib, a selective COX‐2 inhibitor, alleviated acute gout inflammation by blocking prostaglandin E2 (PGE2) synthesis. Notably, COX‐2 (PTGS2) and SOCS3 were co‐upregulated in gout patients and murine models, suggesting their potential as dual biomarkers for gout [2, 35]. The Coremine Medical database identified five *SOCS3* and Gout‐associated traditional Chinese herbs: *Su Tou* (root of *Perilla frutescens* , Lamiaceae), *Bai Su Geng* (stem of *Perilla* species), *Sang Ye* (dried leaf of *Morus alba* , Moraceae), *Sang Zhi* (twig of *Morus alba* ), and *Bai Su Zi* (fruit of *Perilla* species). The extract of *Su Tou* inhibits XOD activity to reduce uric acid production, while components from *Perilla* leaves demonstrate hypouricemic effects in hyperuricemic animal models [36]. *Bai Su Geng* contains phenolics, coumarins, terpenoids, and alkaloids that mitigate gout‐associated inflammation and oxidative stress by suppressing IL‐1β/TNF‐α and scavenging free radicals [37, 38]. *Bai Su Zi* is rich in α‐linolenic acid (ALA, > 65% in *Perilla* seeds), an ω‐3 polyunsaturated fatty acid that alleviates gouty arthritis by modulating lipid metabolism, inhibiting IL‐6/TNF‐α, and promoting anti‐inflammatory mediators like lipoxins [39, 40]. *Perilla* extracts synergize with conventional anti‐gout drugs (e.g., allopurinol) to enhance efficacy and reduce adverse effects via multi‐target mechanisms [41, 42]. *Sang Ye* aqueous extract lowers uric acid by inhibiting hepatic XOD and upregulating renal ABCG2 expression, while suppressing prostaglandin E2 synthesis to attenuate joint inflammation [43, 44]. *Sang Zhi* bioactive components alleviate gouty arthritis by inhibiting NF‐κB signaling to reduce IL‐6/TNF‐α release, thereby ameliorating local inflammation and pain [37, 45].

In summary, our integrated bioinformatics analysis, machine learning approach, and clinical validation have identified *SOCS3* as a pivotal diagnostic biomarker for gout, with its regulatory role intricately linked to PANoptosis ‐ related mechanisms. This work not only provides a novel translational framework for developing diagnostic biomarkers and targeted therapeutics in gout but also advances our understanding of shared pathological mechanisms across inflammatory diseases, offering conceptual inspiration for research on common inflammatory pathways. While this study confirmed the biomarker's relevance through clinical samples, limitations include a limited sample size and the lack of stratification by disease stages. Subsequent studies should employ in vitro and in vivo models to elucidate the mechanistic underpinnings of *SOCS3* in gout pathogenesis. Future research may further explore the roles of these molecular pathways in gout progression, offering novel insights and therapeutic targets for the precise diagnosis and treatment of the disease.

## Author Contributions

J.C.: data curation, methodology, validation, writing – original draft, writing – review and editing. A.L.: data curation, validation, writing – review and editing. G.L.: writing – review and editing. Z.W.: data curation, writing – review and editing. Y.L.: resources, writing – review and editing.

## Conflicts of Interest

The authors declare no conflicts of interest.

## Supporting information


**Figure S1.** The flowchart of this study.

**Figure S2.** Enrichment analysis of the common genes of PANoptosis and gout. (A) KEGG pathway analysis of the common genes. Different colors represent various significant pathways and related enriched genes. (B, C, D) GO analysis of the common genes, including biological process, cellular component, and molecular function, respectively.

**Figure S3.** Expression of *SOCS3* in clinical samples.

**Table S1.** The primers used in this study.

**Table S2.** Prediction of potential therapeutic drugs from DsigDB.

**Table S3.** Prediction of potential therapeutic drugs from Coremine database.

## Data Availability

The data that support the findings of this study are available from the corresponding author upon reasonable request.
