# Supplementary figures and images for: Decoding PANoptosis in Gout: Signature Gene Identification and Immune Infiltration Profiling
Source: Int J Rheum Dis. 2025 Jul 4;28(7):e70344. doi: 10.1111/1756-185X.70344 (PMC12231154; doi:10.1111/1756-185X.70344)

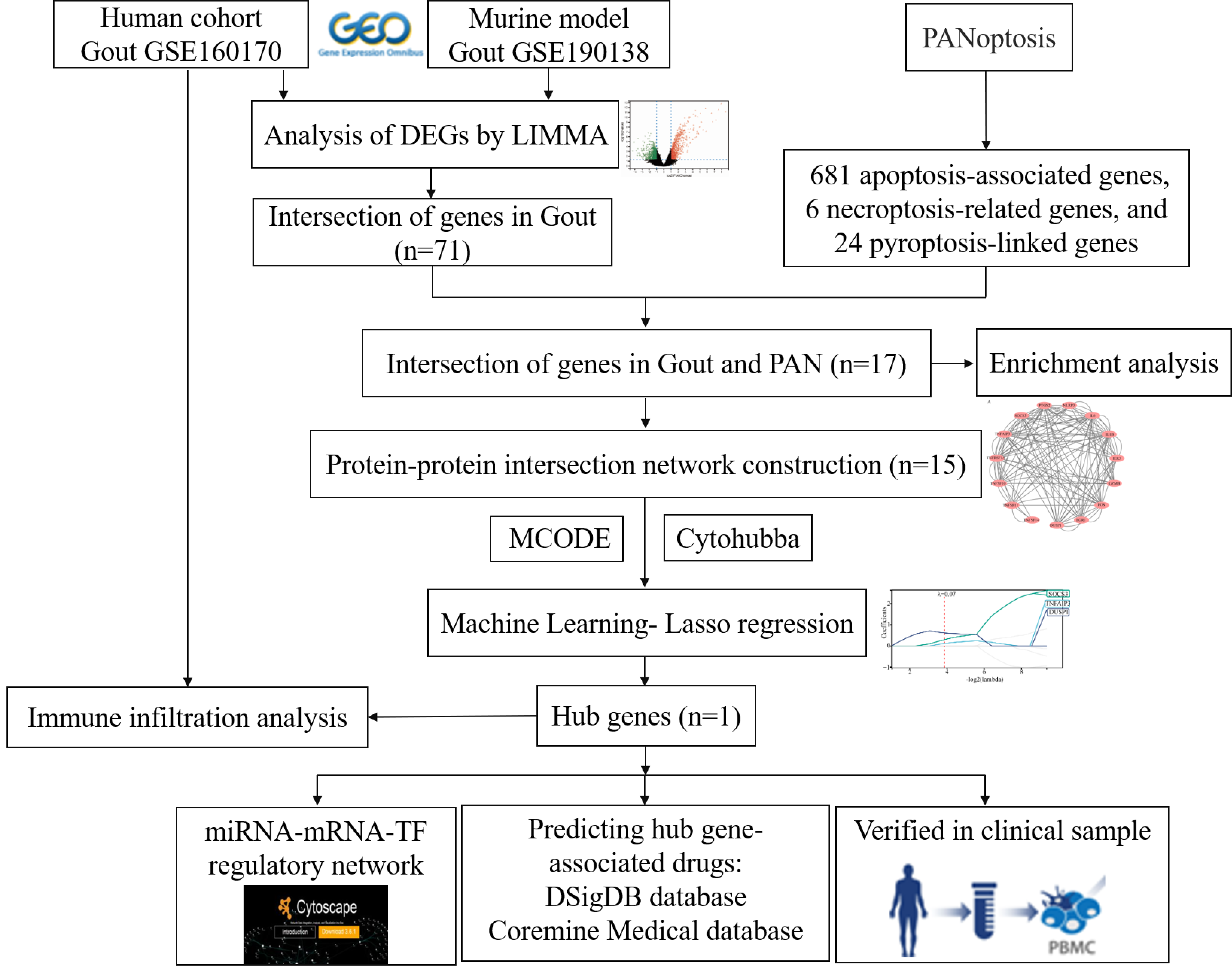

Supplement: Supplementary file 1 — Figure S1. The flowchart of this study. [file APL-28-e70344-s005.tif]

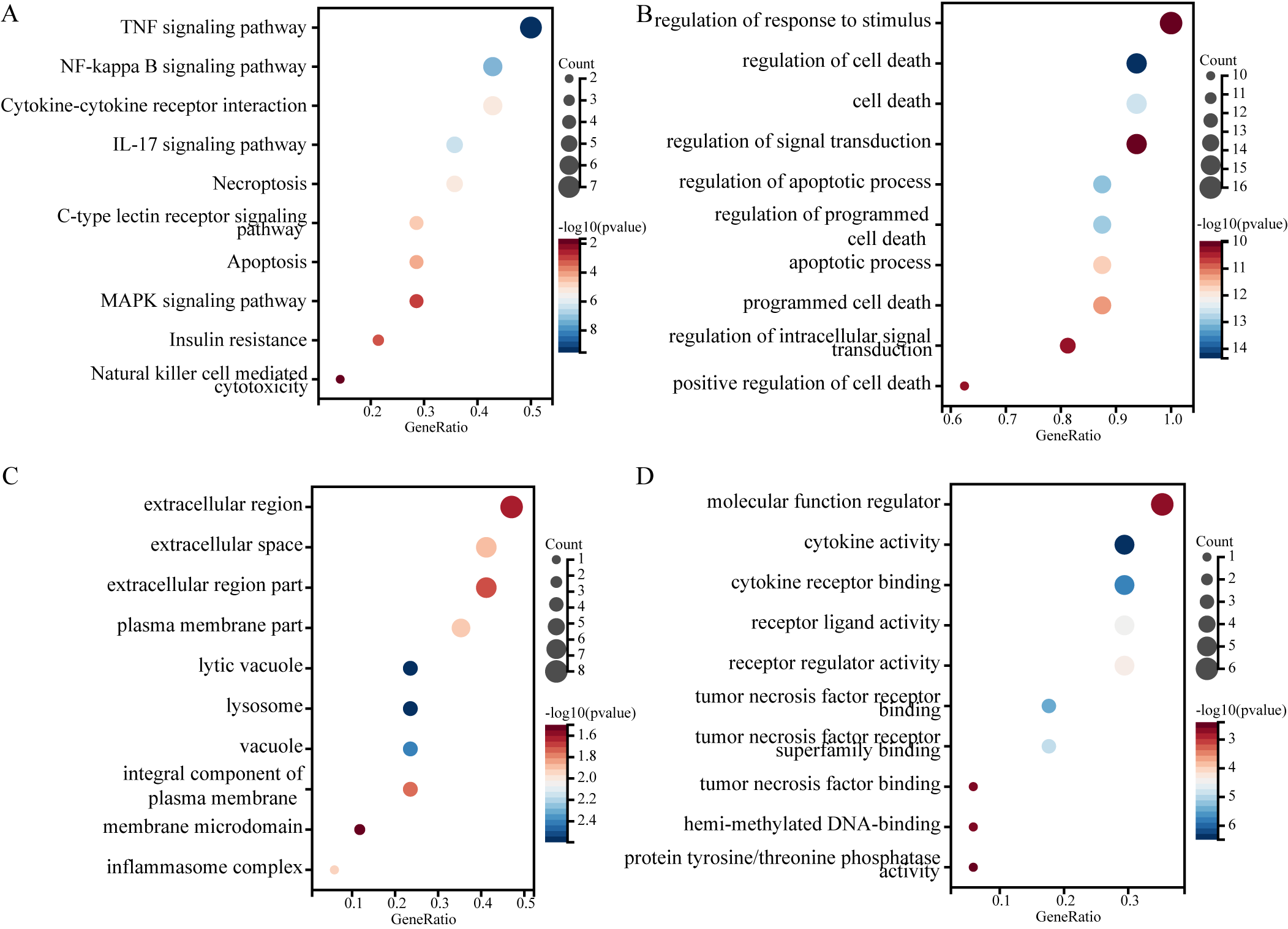

Supplement: Supplementary file 2 — Figure S2. Enrichment analysis of the common genes of PANoptosis and gout. (A) KEGG pathway analysis of the common genes. Different colors represent various significant pathways and related enriched genes. (B, C, D) GO analysis of the common genes, including biological process, cellular component, and molecular function, respectively. [file APL-28-e70344-s004.tif]

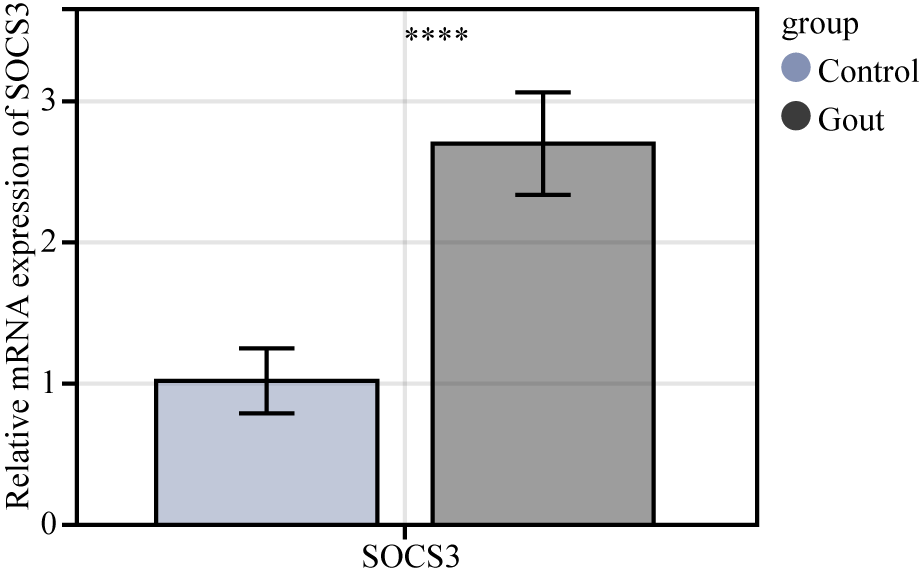

Supplement: Supplementary file 3 — Figure S3. Expression of SOCS3 in clinical samples. [file APL-28-e70344-s001.tif]
